# Three‐dimensional ultrasonography could be a potential non‐ionizing tool to evaluate vertebral rotation of subjects with adolescent idiopathic scoliosis

**DOI:** 10.1002/jsp2.1259

**Published:** 2023-05-08

**Authors:** Tin Yan Lee, De Yang, Kelly Ka‐Lee Lai, Rene M. Castelein, Tom P. C. Schlosser, Winnie Chu, Tsz‐Ping Lam, Yong‐Ping Zheng

**Affiliations:** ^1^ Department of Biomedical Engineering The Hong Kong Polytechnic University Hong Kong Hong Kong; ^2^ Research Institute for Smart Ageing The Hong Kong Polytechnic University Hong Kong Hong Kong; ^3^ Department of Orthopaedic Surgery University Medical Center Utrecht Utrecht The Netherlands; ^4^ Department of Imaging and Interventional Radiology The Chinese University of Hong Kong Hong Kong SAR China; ^5^ SH Ho Scoliosis Research Lab, Joint Scoliosis Research Center of the Chinese University of Hong Kong and Nanjing University, Department of Orthopaedics & Traumatology, The Chinese University of Hong Kong Hong Kong Hong Kong

**Keywords:** adolescent idiopathic scoliosis, laminae, nonionizing axial, three‐dimensional ultrasonography, vertebral rotation

## Abstract

**Background:**

Three‐dimensional (3D) ultrasonography is nonionizing and has been demonstrated to be a reliable tool for scoliosis assessment, including coronal and sagittal curvatures. It shows a great potential for axial vertebral rotation (AVR) evaluation, yet its validity and reliability need to be further demonstrated.

**Materials and Methods:**

Twenty patients with adolescent idiopathic scoliosis (AIS) (coronal Cobb: 26.6 ± 9.1°) received 3D ultrasound scan for twice, 10 were scanned by the same operator, and the other 10 by different operators. EOS Bi‐planar x‐rays and 3D scan were conducted on another 29 patients on the same day. Two experienced 3D ultrasonographic researchers, with different experiences on AVR measurement, evaluated the 3D ultrasonographic AVR of the 29 patients (55 curves; coronal Cobb angle: 26.9 ± 11.3°). The gold standard AVR was determined from the 3D reconstruction of coronal and sagittal EOS radiographs. Intra‐class correlation coefficients (ICCs), mean absolute difference (MAD), standard error measurements (SEM), and Bland–Altman's bias were reported to evaluate the intra‐operator and inter‐operator/rater reliabilities of 3D ultrasonography. The reliability of 3D ultrasonographic AVR measurements was further validated using inter‐method with that of EOS.

**Results:**

ICCs for intra‐operator and inter‐operator/rater reliability assessment were all greater than 0.95. MAD, SEM, and bias for the 3D ultrasonographic AVRs were no more than 2.2°, 2.0°, and 0.5°, respectively. AVRs between both modalities were strongly correlated (*R* ^2^ = 0.901) and not significantly different (*p* = 0.205). Bland–Altman plot also shows that the bias was less than 1°, with no proportional bias between the difference and mean of expected and radiographic Cobb angles.

**Conclusion:**

This study demonstrates that 3D ultrasonography is valid and reliable to evaluate AVR in AIS patients. 3D ultrasonography can be a potential tool for screening and following up subjects with AIS and evaluating the effectiveness of nonsurgical treatments.

## Graphical abstract

3D ultrasonography is valid and reliable to evaluate AVR in adolescent idiopathic scoliosis (AIS) patients, and could be a potential tool for screening and following up subjects with AIS and evaluating the effectiveness of nonsurgical treatments.

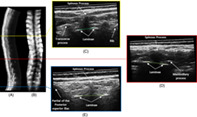

## 1 INTRODUCTION

Adolescent idiopathic scoliosis (AIS) is a three‐dimensional (3D) deformity. Other than the deformities in the coronal and sagittal planes, axial vertebral rotation (AVR) is a key characteristic in scoliosis evaluation^1^ and a known risk factor on curve progression.^2^ The effectiveness of non‐surgical treatments, such as brace application and scoliosis‐specific exercise, is commonly evaluated by their abilities on vertebral rotation correction.^3^, ^4^, ^5^ Therefore, a reliable tool that provides accurate AVR measurement is essential for curve progression management.

Scoliometer, a convenient tool used for scoliosis screening in usual daily clinical practise, is an inclinometer applied to the back of a subject in forward bending postured to assess the rotation of the chest wall.^6^ However, this approach does not provide direct measurement of vertebral rotation and the difference in rotation between forward bending and the standing position is unknown.^7^ Various imaging modalities have been used to evaluate AVR for the past decades. The first radiographic study to evaluate AVR was conducted by Cobb, using the relative position of the spinous process to the vertebrae body for grading,^8^ followed by other studies utilizing different landmarks such as pedicles for the evaluation of AVR.^9^, ^10^ Nevertheless, these measurements are on projected features of 2D coronal images, not directly on the transverse plane. Nowadays, the EOS radiography system allows for 3D spinal reconstructions based on simultaneously acquired biplanar, 2D radiographs. By manual matching of reference landmarks, a virtual 3D spinal alignment model is created on which AVR could be measured. While the radiographs are obtained low‐dose and AVR can be evaluated with high reliability^11^ and is considered the “gold standard,” the process of 3D reconstruction is time‐consuming and the apparatus is relatively costly. Furthermore, despite the limited ionizing radiation, it is not allowed for; for example the serial assessment of spinal dynamics in various body positions or assessment of asymptomatic children. Investigation of AVR can also be achieved using computed tomography (CT) and magnetic resonance imaging (MRI). While CT and MRI allow precise measurement of AVR,^12^, ^13^ they are obtained in non‐weightbearing position and CT requires the usage of a relative high dosage of radiation.^14^, ^15^, ^16^

Currently, 3D ultrasonography has gained popularity of evaluating spinal alignment and scoliosis.^17^, ^18^, ^19^, ^20^, ^21^ The advantages of 3D ultrasonography imaging are its nonionizing, readily accessible, and low‐cost nature. During the past decade, a 3D ultrasonography system has been developed and demonstrated to provide valid and reliable measurements to access coronal^17^, ^19^, ^20^, ^21^, ^22^ and sagittal^23^, ^24^, ^25^ curvatures using various landmarks of the posterior spinal elements such as spinous and transverse processes and laminae. Evaluation of AVR could also be assessed using these landmarks.^26^ However, the reliability of this 3D ultrasonography system to measure AVR has yet to be investigated.

Therefore, the objective of this study was to determine the validity and reliability of the apical AVR measurement acquired with a 3D ultrasonography system by comparing the results with that obtained from the EOS system.

## 2 MATERIALS AND METHODS

### 2.1 Subjects

Ethical approval was given from the local ethical review boards and informed consent was obtained from all subjects. The inclusion criteria of the subjects involved in this study were: (1) diagnosed with AIS; (2) without spinal implants; (3) Cobb angle ranges from 10 to 60°; and (4) removal of braces 48 h prior to x‐ray imaging for those who were receiving brace treatment. All patients were recruited from the Department of Orthopedics and Traumatology of a local university and were screened and diagnosed as AIS by an experienced medical doctor in the department.

### 2.2 Data acquisition

To evaluate the scan–rescan reliability of 3D ultrasonography on AVR assessment, 20 subjects with AIS were required to receive 3D ultrasonography scan for twice. Ten of them were scanned by the same experienced operator, Operator 1; whereas another 10 were scanned by Operator 1 and Operator 2, another operator who has similar experience in 3D ultrasound scanning. Additional 30 subjects were recruited to evaluate the intra‐rater and inter‐rater reliability and validity of 3D ultrasonography on AVR assessment. These subjects received both x‐ray imaging and 3D ultrasonography scanning on the same day, first radiographs, followed by ultrasound assessment.

Low‐dose bi‐planar x‐rays were simultaneously captured by the EOS 2D/3D system® (EOS imaging, Paris, France).^27^ Subjects were required to stand naturally with extended hips and knees and with hands on a support about 1.7 m from ground level. The 3D ultrasonography was conducted manually by one trained operator using a customized 3D ultrasonography system, which consists of a linear ultrasound probe with central frequency of 7.5 MHz and 7.5 cm width, equipped with an electromagnetic spatial sensor to record the position and orientation of the probe, and a scanning time of approximately 30 s. Subjects underwent ultrasound scanning in a natural standing posture with arms resting by their sides. The ultrasonography system also contains adjustable supporters at the level of the anterior superior iliac spines and clavicles, facilitating subjects with different sizes to minimize the motion induced by the posterior pressure of the ultrasound transducer.^21^ Detailed specification and the testing protocol of the 3D ultrasound imaging system and the testing protocol were reported in previousstudies.^19^, ^24^

### 2.3 Apical vertebral rotation measurements

**FIGURE 1:**
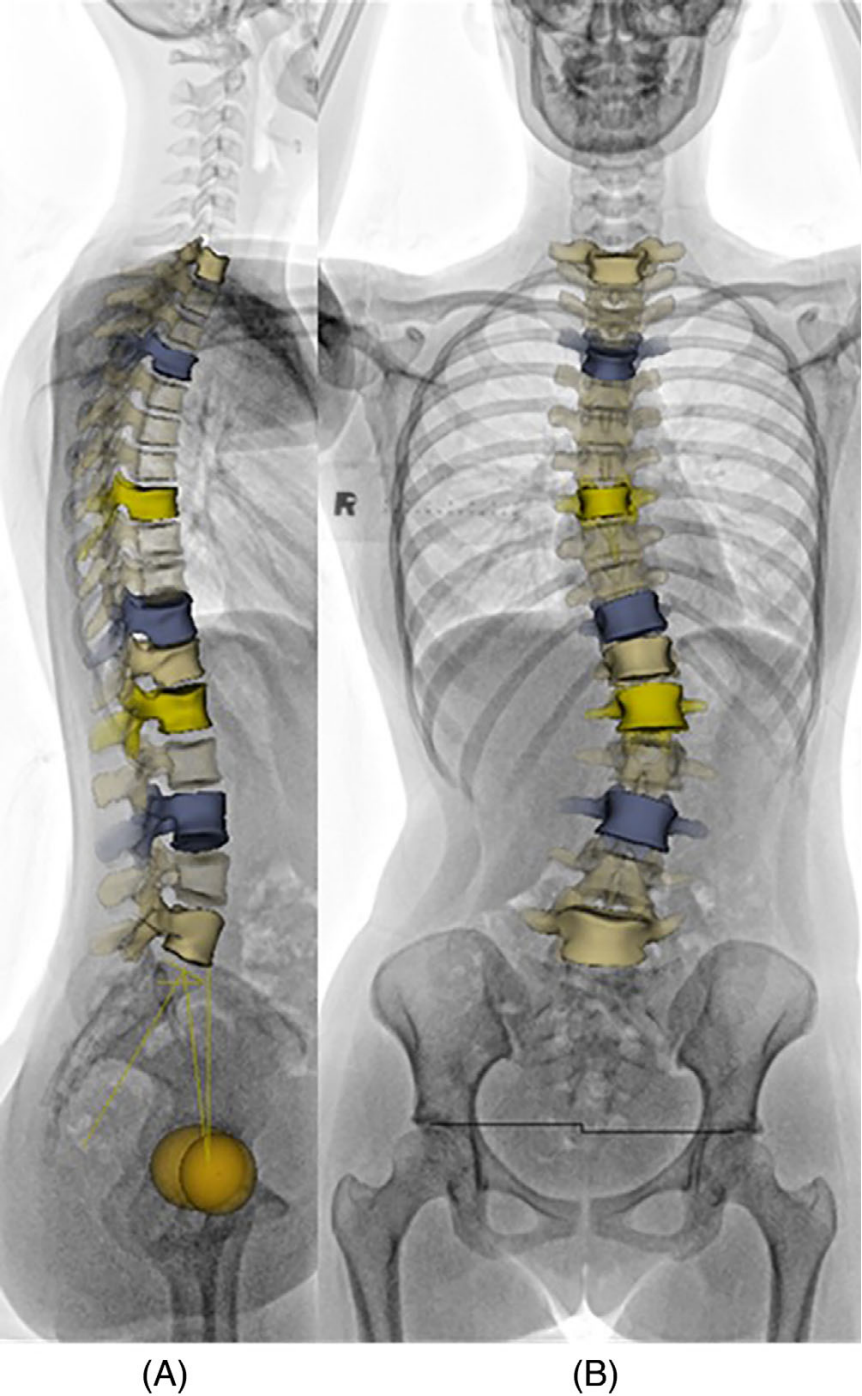
EOS full spine image of a patient with adolescent idiopathic scoliosis in the (A) coronal and (B) sagittal plane together with the three‐dimensional (3D) reconstruction of the spine from T1 to L5 vertebrae. The upper and lower end vertebrae were illustrated in blue color and the vertebrae with largest axial vertebrae rotation for each curve were illustrated in light yellow.

3D spinal reconstruction was acquired using sterEOS software, which requires manual mapping of the thoracic and lumbar vertebrae contour from T1 to S1 on the coronal and sagittal x‐ray images (Figure 1A,B).^28^ After reconstruction, the global axial rotation of each vertebra can be obtained. AVR of each vertebra was then computed by measuring the angle of rotation between the vertebra and the hip‐axis in the horizontal plane. The AVR that possess the maximum value in each curve, either major or compensatory curve, was considered as the apical AVR. While apical vertebrae of major curves are mostly automatically identified by the software, the apical vertebrae of compensatory curves would generally require manual identification.

**FIGURE 2:**
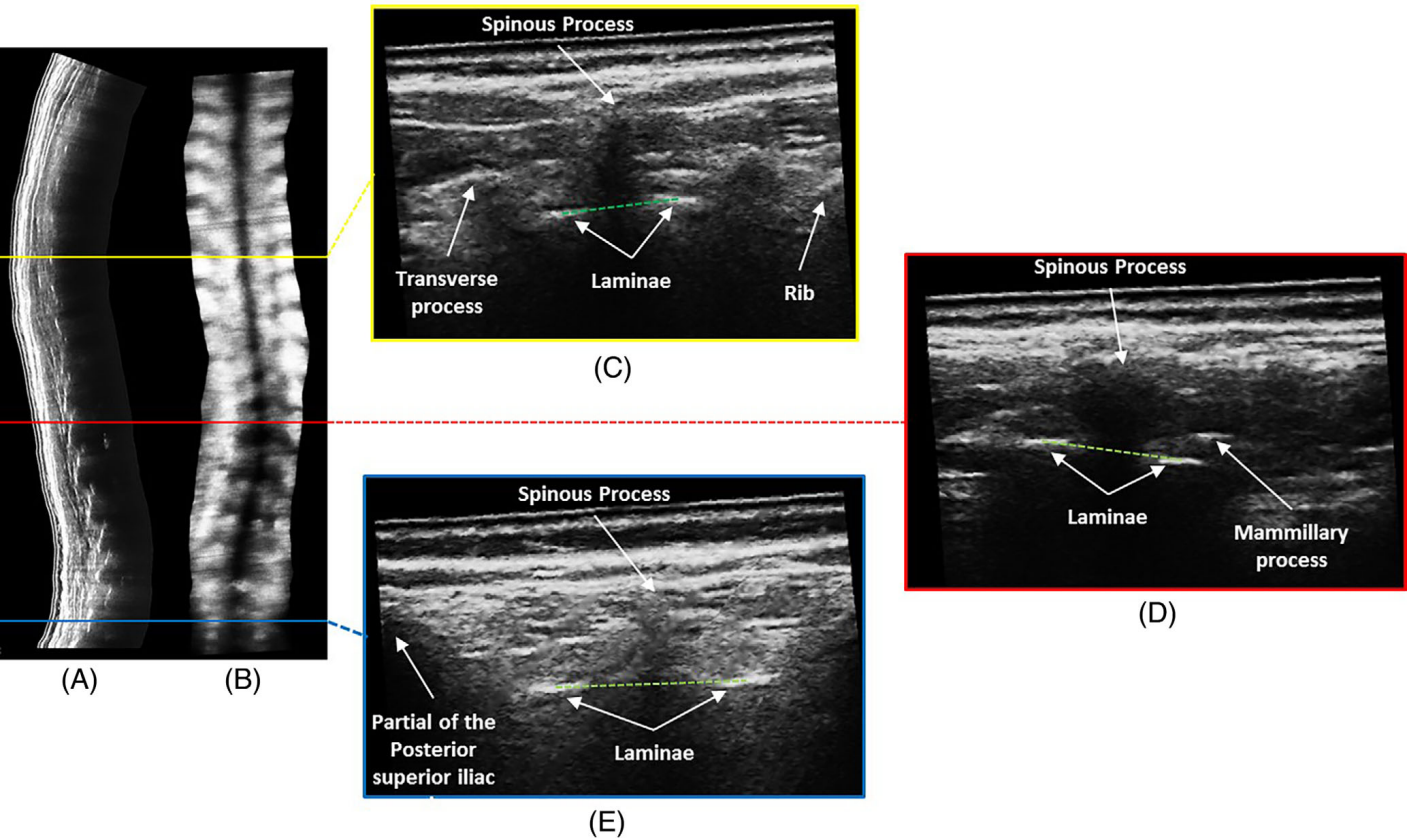
The reconstructed 3D ultrasonography images of a spine from a subject with AIS in (A) sagittal plane; (B) coronal plane; transverse planes of (C) T8, (D) L1, and (E) S1. The green dotted line joins the centre of laminae of the three regions which was used to obtain the corresponding rotation in order to evaluate the axial rotation angle.

For AVR measurement on ultrasound images, the 3D ultrasound volume captured was transferred to a customized software to reconstruct the orthogonal ultrasound images. With the aid of the coronal and sagittal view of the ultrasound images, the bilateral laminae were manually identified on the true transverse ultrasound frames using a customized software. The apical AVR was defined as the angle between the line connecting the centers of the bilateral laminae (CoL) of the apical vertebra and of S1 was defined as the apical AVR.^29^ Apical AVR measurements on the 3D ultrasound images were performed at the same spinal level and the level with maximum AVR on the EOS reconstructions (Figure 2).

Two raters were involved for the ultrasound measurements. One of the raters, R1, also performed AVR acquisition from EOS using sterEOS software, who has 7 years of experience of scoliosis research using 3D ultrasonography and has been well trained to generate 3D bi‐planar x‐ray images for more than 1 year. R1 was responsible to compile the measurement for intra‐operator and inter‐operator reliability and validity of 3D ultrasonography. Another rater, R2, has more than 2‐year experience in studying scoliosis classification using 3D ultrasonography, but no experience in AVR measurement before this study. Both raters worked independently to select the transverse image reconstruction and measure AVR angles for the evaluation of intra‐rater and inter‐rater reliability of 3D ultrasonography. AVR angles were measured from the same 3D ultrasonography scan twice by both raters, with an interval of 1 week to minimize the bias.

### 2.4 Statistical analysis

Statistical analyses were conducted using SPSS version 26.0 (IBM, SPSS Inc.). Intra‐class correlation coefficient (ICC) (3,1) with 95% confidence intervals (two‐way mixed and absolute agreement) were used to assess the intra‐operator and intra‐rater reliability of 3D ultrasonography measurements, while ICC (2,1) with 95% confidence intervals (two‐way random and absolute agreement) were used to assess the inter‐operator and inter‐rater reliability between 3D ultrasonography and EOS measurements. The inter‐method reliability was assessed using ICC (2,1) with 95% confidence intervals (two‐way random and absolute agreement).^29^ Currier criteria were adopted for the ICC values: excellent (0.90–1.00), good (0.80–0.89), fair (0.70–0.79), and poor (<0.60).^30^ Mean absolute difference (MAD), standard error measurement (SEM), measurement bias and limits of agreements between operators and raters were also evaluated for all assessments. The agreement of the AVR measurements obtained from the two modalities was demonstrated in Bland–Altman plot and the corresponding correlation was evaluated by Pearson correlation. Paired t‐test was further conducted to compare the AVRs AVR measurements obtained from the two modalities. Post hoc linear regression between the difference and the mean of 3D ultrasonographic and EOS AVR angles were performed to investigate whether there is proportional bias or not. The level of significance was set at 0.05.

## 3 RESULTS

A total of 17 curves from 10 AIS subjects (Age: 15.5 ± 1.6 years; Cobb: 24.7 ± 9.5°) were involved to evaluate the intra‐operator reliability of 3D ultrasonography, whereas a total of 19 curves from 10 AIS subjects (Age: 15.1 ± 1.2 years; Cobb: 28.4 ± 8.5°) were involved to evaluate the inter‐operator reliability of 3D ultrasonography. To evaluate the intra‐rater and inter‐rater reliability and validity, 30 patients with AIS were recruited. But since the reflection of the lamina on the concave side from a subject with a lumbar curve of Cobb angle >50° could not be observed in 3D ultrasonography, only 29 subjects (Age: 15.5 ± 1.7 years; Cobb: 26.9 ± 11.6°) were ultimately included. Table 1 summarized the demographic information of all the subjects.

**TABLE 1:** Demographic information of the patients.

	Intra‐operator reliability	Inter‐operator reliability	Intra‐rater/inter‐rater reliability and validation
Age^a^	15.7 ± 1.6	15.1 ± 1.2	15.5 ± 1.7
Gender	7 F	8 F	19 F
	3 M	2 M	10 M
Body mass index (kg/$m^{2}$)^a^	18.0 ± 2.0	18.4 ± 1.2	18.2 ± 2.1
Coronal Cobb [range] (°)^a^	24.7 ± 9.5 [13–49]	28.4 ± 8.5 [18–48]	26.9 ± 11.3 [12–55]
Total number of curves	17	19	55
Curve type	3 single [Rt T]	1 single [Lt L]	4 single [Lt L]
	6 double [Rt T, Lt (T)L]	7 double [Rt T, Lt (T)L]	1 single [Rt T]
	1 others [Lt PT + Rt T]	1 double [Lt T, Rt (T)L]	18 double [Rt T, Lt (T)L]
		1 others [Lt PT + Rt T]	2 triple [Lt PT, Rt T, Lt (T)L]
			4 Others [Lt PT + Rt T]

Abbreviations: L, lumbar; Lt, left; PT, proximal thoracic; Rt, right; T, thoracic.

^a^ Data are mean ± standard deviation.

The time required for apical AVR acquisition for 3D ultrasonography was average 3 min, whereas the time required for EOS 3D spinal reconstructions and AVR measurement was on average 20 min.

### 3.1 Intra‐operator and inter‐operator reliabilities

Table 2 demonstrates the intra‐operator and inter‐operator reliabilities for 3D ultrasonographic AVR measurements conducted by R1. The ICC values, mean MAD, and SEM were (0.975, 1.6°, 1.4°) for scans from the same operator and (0.969, 2.2°, 1.7°) for scans from different operator, respectively. Bland–Altman analysis showed that AVR measurement bias obtained from consecutive scans were less than 0.5°.

**TABLE 2:** Intra‐operator and inter‐operator variation and reliability for 3D ultrasound axial vertebrae rotation angles.

	Number of curves	ICC (95% CI)	MAD (°)	SEM (°)	Bias ± limits of agreement (°)
Intra‐operator	17	0.975 (0.933 – 0.991)	1.6 (0.3 – 4.3)	1.4	0.0 ± 4.0
Inter‐operator	19	0.969 (0.923 – 0.988)	2.2 (0.4 – 3.8)	1.7	0.4 ± 4.8

Abbreviations: CI, confidence interval; ICC, Intra‐class correlation coefficient; MAD, mean absolute difference; SEM, standard error of measurement.

### 3.2 Intra‐rater and inter‐rater reliabilities

Tables 3 and 4 demonstrate the intra‐rater and inter‐rater reliabilities for 3D ultrasonographic AVR measurements conducted by R1 and R2, respectively. For intra‐rater test, the ICC values, mean MAD, and SEM were (0.987, 1.2°, 1.1°) for R1 and (0.969, 1.9°, 1.6°) for R2, respectively. Bland–Altman analysis showed that AVR measurement bias obtained between consecutive measurement were less than 0.5° for both raters. For inter‐rater test, the ICC values, mean MAD, and SEM of the measurements of two raters were (0.953, 2.2°, 2.0°). Bland–Altman analysis showed that measurement bias obtained between the AVR measurements of the raters were about 0.5°.

**TABLE 3:** Intra‐rater variation and reliability for 3D ultrasound axial vertebrae rotation angles.

	Number of curves	ICC (95% CI)	MAD (°)	SEM (°)	Bias ± limits of agreement (°)
Rater 1	55	0.987 (0.978 – 0.992)	1.2 (0.0 – 4.6)	1.1	0.3 ± 3.0
Rater 2	55	0.969 (0.947 – 0.982)	1.9 (0.0 – 7.5)	1.6	−0.2 ± 4.5

Abbreviations: CI, confidence interval; ICC, intra‐class correlation coefficient; MAD, mean absolute difference; SEM, standard error of measurement.

**TABLE 4:** Inter‐rater variation and reliability for 3D ultrasound axial vertebrae rotation angles.

	Number of curves	ICC (95% CI)	MAD (°)	SEM (°)	Bias ± limits of agreement (°)
Rater 1 vs. Rater 2	55	0.953 (0.921–0.972)	2.2 (0.2 – 9.7)	2.0	0.5 ± 5.6

Abbreviations: CI, confidence interval; ICC, Intra‐class correlation coefficient; MAD, mean absolute difference; SEM, standard error of measurement.

### 3.3 Validity

**FIGURE 3:**
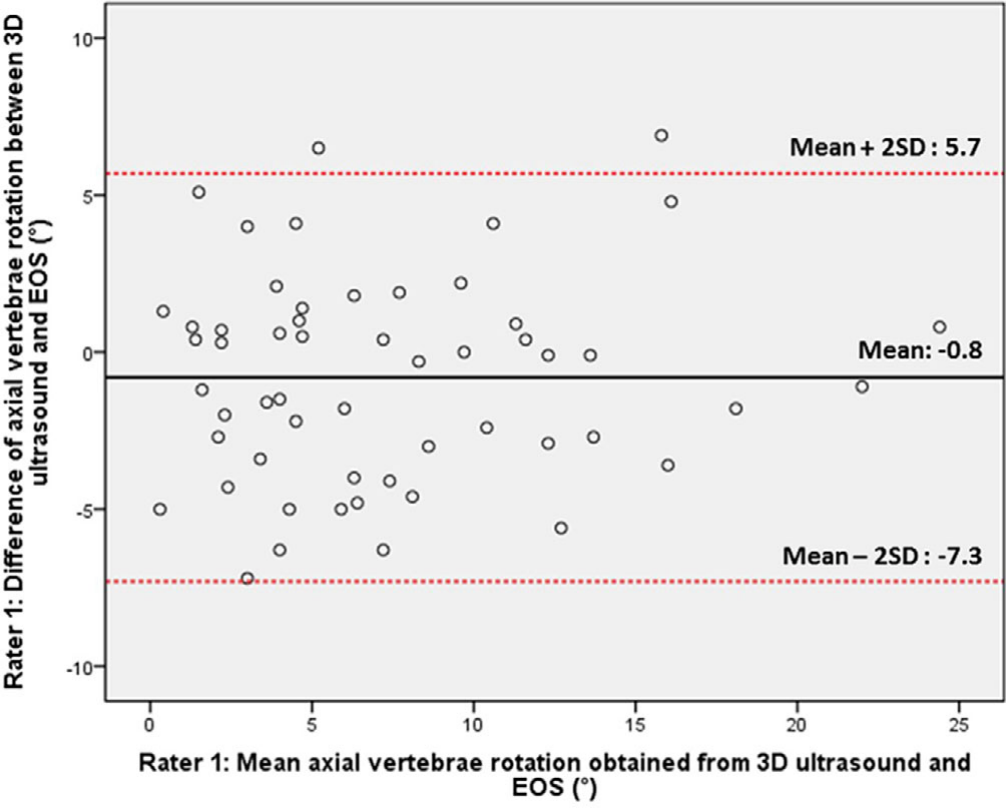
The Bland–Altman plot from rater 1's result: comparing the absolute value of axial vertebra rotation measurement between 3D ultrasonography and EOS.

Table 5 shows the inter‐method (3D ultrasonography versus EOS) reliability for AVR measurements. The ICC values, mean MAD, and SEM of the two measurements were (0.930, 2.7°, 2.4°). Bland–Altman plot showed that AVR measurement bias obtained between two modalities were less than 1° (Figure 3). In addition, no significant correlation was found between the difference and mean of expected and radiographic Cobb angles for (*p* = 0.521), indicating that there was no proportional bias, that is, curve severity did not influence the amount of variation.

**TABLE 5:** Inter‐method variation and reliability between axial vertebrae rotation angles obtained from 3D ultrasound and EOS by Rater 1.

	Number of curves	ICC (95% CI)	MAD (°)	SEM (°)	Bias ± limits of agreement (°)
3DUS vs. EOS	55	0.930 (0.883 – 0.959)	2.7 (0.0 – 7.2)	2.4	−0.8 ± 6.5

Abbreviations: 3DUS, three‐dimensional ultrasound; CI, confidence interval; ICC, Intra‐class correlation coefficient; MAD, mean absolute difference; SEM, standard error of measurement.

**FIGURE 4:**
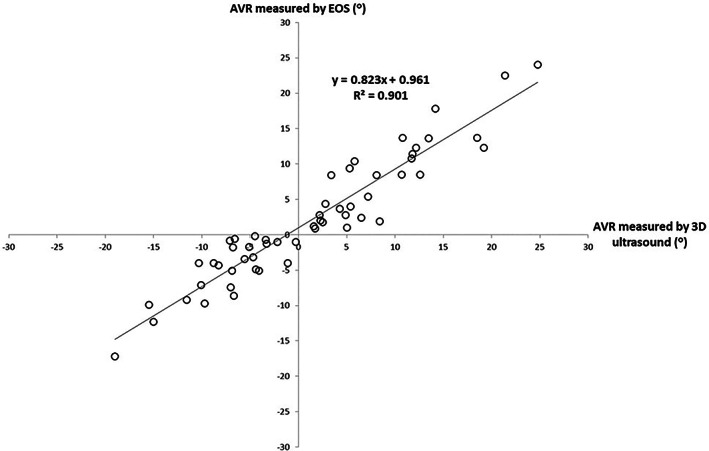
Correlation of AVR measurements by rater 1 using 3D ultrasonography versus that of EOS. Positive AVR corresponds to anti‐clockwise vertebrae rotation from the craniocaudal view; whereas negative AVR corresponds to clockwise vertebrae rotation from the craniocaudal view. AVR, axial vertebrae rotation; 3D, three dimensional.

No significant difference was observed between the AVRs (*p* = 0.205). The Pearson correlation plot showed that high correlation ($R^{2}$ = 0.901) between the AVR measurements was obtained from 3D ultrasonography and EOS assessment (Figure 4).

## 4 DISCUSSION

Through this study, good agreement was achieved between the AVR measurements using 3D ultrasonography and EOS and excellent intra‐ and inter‐reliabilities were demonstrated in AVR measurements using 3D ultrasonography in the upright position. This justified the customized 3D ultrasonography system and the compatible 3D software could provide reliable and valid AVR measurements for patients with AIS.

Since 3D ultrasonography scan and AVR measurements were all conducted manually, AVR evaluation could be affected between scans of operators and raters. ICCs for intra‐operator and inter‐operator/rater reliability assessment were all greater than 0.95, demonstrating high reliability for 3D ultrasonography on AVR measurement. Bland–Altman analysis shows that biases between AVR measurements obtained from scans conducted by either the same or different operators were no more than 0.5°, with comparative MAD and SEM no more than 2.2° and 1.7°, respectively. This demonstrated that the AVR measurements obtained from different 3D ultrasound scans are reliable. Though MAD of 2.2° was observed between the AVR measurements generated by the experienced and novice rater, the difference is within clinically accepted value and the ICCs were larger than 0.9. This demonstrated that reliable AVR measurements could be achieved using the customized 3D software, regardless of the experience of the rater. Higher reliabilities and smaller MAD were observed for the AVR measurement by the more experienced rater from the intra‐rater and validity analysis, which is expected as 3D ultrasonographic measurement accuracy is highly related to the experience of the rater.^31^ The MAD and the $R^{2}\;$correlation between the AVR measurements of two imaging modalities by R1 was 2.7° and 0.901, stating that the difference between two methods was clinically acceptable and the two values are highly correlated. Bias of less than 1° and absence of proportional bias were observed from the Bland–Altman plot in this study, which indicated that AVRs obtained from 3D ultrasonography and EOS have good agreement and the severity of vertebrae rotation has no effect on the variation of the differences between the AVR measurements of the two modalities.

Various studies have evaluated AVR using 3D ultrasonography using phantoms^32^, ^33^ or patients with AIS.^34^, ^35^ Yet, only one study evaluated AVR on patients with AIS in upright postures,^35^ which is considered to be the functional posture without altering the natural spine curvature. In addition, none of these studies reported the operator reliabilities on AVR measurement using 3D ultrasound. The ICC values of this study (intra‐rater: 0.987 and 0.969 for R1 and R2, respectively, inter‐rater: 0.953) were comparable to all these studies (intra‐rater ICC ranges from 0.95 to 1.00 and inter‐rater ICC ranges from 0.91 to 0.98).^32^, ^33^, ^34^, ^35^ In addition, the MAD and Bland–Altman bias values in this study (Intra‐rater: 1.2–1.9°, inter‐rater: 2.2°, bias: −0.2–0.5°) are comparable to those by Trac et al.^35^ Furthermore, our inter‐method MAD and bias were found to be smaller. Therefore, this is the first comprehensive study that demonstrates 3D ultrasound could provide reliable and repeatable AVR assessments in upright postures, without systematic difference within and between operators and raters.

Other than evaluating the deformities in the transverse, coronal, and sagittal planes demonstrated in this study and in previous studies,^19^, ^20^, ^21^, ^22^, ^23^, ^24^ the capability of the 3D ultrasonography system used in this study has been further exploited to investigate its ability on evaluating the coupling effect between coronal and sagittal curvatures^36^ and classification of mild scoliosis.^37^ Recently, a portable version of the 3D ultrasonography system used in this study was developed.^38^ The results demonstrated in this study suggested that 3D ultrasonography could be a potential tool for medical professionals to conduct on‐site AVR evaluation in any places which ultimately serves the purpose of AIS screening and diagnosis. It should be noted that natural standing posture was adopted for 3D ultrasonography scanning in this study, because the ultrasonography system comes with frontal supporters at the clavicle and anterior superior iliac spine level, which provides extra stabilization for the subjects during assessment. Therefore, a posture that could provide stabilization to the subject at the same time generate comparable AVR results with that obtained from radiograph should be designed and adopted.

With the enhancement of imaging technology, 3D reconstruction of the spine could be achieved from anterior–posterior and lateral EOS images using SterEOS software.^39^ Although the 3D reconstruction data could be used to conduct various applications such as monitoring curve progression^40^ or classification of AIS^41^ more effectively; it should be noted that the EOS reconstructions are based on the template information of the EOS system, but not the real spinal image itself. Furthermore, it should be noted that acquisition of AVR from EOS is also a manual process, and therefore the measurement is also subjected to error due to variations of the observed vertebral anatomy. Nevertheless, both 3D spine reconstruction from EOS radiographs and acquisition of spinal parameters from 3D ultrasonography involve intensive training and knowledge about the anatomy of spine that are time consuming. Application of deep learning using neural network could be a potential solution to make these processes more effective in clinical routine in future studies.^42^, ^43^

Small sample size was one of the limitations of this study. In addition, although AVR severity was demonstrated to have no significant effect on the variation of the differences between the 3D ultrasonography and EOS AVR measurements, the curves involved in this study were not large enough (12.0–55.3°); therefore, the applicability of 3D ultrasonography to evaluate AVR of patients with severe scoliosis is yet to be investigated. Indeed, this study is proposing 3D ultrasonography to be a complementary imaging modality to evaluate curve progression and non‐operative treatment such as brace and specific scoliosis exercise, for patients with mild and moderate AIS, but not a replacement of conventional imaging modalities, such as x‐ray and CT. For surgical planning and flexibility evaluation, x‐ray and CT are more preferable. One subject with Cobb angle larger than 50° and AVR larger than 25° was excluded, because the lamina on the concave side could not be observed from the transverse ultrasound image. This is due to the limitation of the ultrasonographic technology, as higher the vertebrae rotation, the higher possibility the spinous process would hinder the ultrasound from reaching the lamina. Nevertheless, laminae are still the most preferable posterior landmarks for AVR measurement using 3D ultrasonography, because spinous processes are more subjected to deformity in severe scoliosis, and transverse processes in the respective thoracic and lumbar regions are often interfered by ribs and are not captured by the ultrasound probe since their distance is further than bilateral laminae.

Furthermore, it should be noted that the comparison of the AVR values between two modalities was sustained to inherent discrepancies such as different anatomical structures used for data acquisition. Though bias would be induced, apical AVR measurements were conducted on the same level as a precautionary measure from further inducing error, in order to truly investigate the validity of the AVR assessed by 3D ultrasonography. In future study, the effect of total blindness on AVR measurements and decision making for selection apical vertebrae between the two modalities should be investigated. The learning curve for AVR measurement using 3D ultrasonography should also be explored.

## 5 CONCLUSION

This study demonstrated the AVR measurement using the customized 3D ultrasonography system with 3D analysis software is reliable and validated with that generated from EOS. It is worthwhile to study whether this technique can be used for patients with larger AVR by recruiting more severe scoliosis cases in future study.

## CONFLICT OF INTEREST STATEMENT

Y.P. Zheng reports his role as a consultant to Telefield Medical Imaging Limited for the development of Scolioscan, outside the submitted work and he is the inventor of a number of patents related to 3D ultrasound imaging for scoliosis, which has been licensed to Telefield Medical Imaging Limited through Hong Kong Polytechnic University. He is also a director and shareholder of this startup company. All the other author(s) have no conflicts of interest relevant to this article.
